# Purpura Fulminans and Septic Shock due to Capnocytophaga Canimorsus after Dog Bite: A Case Report and Review of the Literature

**DOI:** 10.1155/2018/7090268

**Published:** 2018-08-26

**Authors:** Elena Mantovani, Stefano Busani, Emanuela Biagioni, Claudia Venturelli, Lucia Serio, Massimo Girardis

**Affiliations:** ^1^Intensive Care Medicine, University Hospital of Modena, Modena, Italy; ^2^Laboratory of Microbiology, University Hospital of Modena, Modena, Italy

## Abstract

Primary infection by* Capnocytophaga canimorsus* after dog bite is rare but may be difficult to identify and rapidly lethal. We describe a case of fatal septic shock with fulminant purpura occurred in a patient without specific risk factor two days after an irrelevant dog bite. The patient was brought to hospital because of altered mental status, fever, and abdominal pain. In a few hours patient became hypoxic and cyanotic. The patient became extremely hypotensive with shock refractory to an aggressive fluid resuscitation (40 ml/kg crystalloids). She received vasoactive drugs, antibiotic therapy, and blood purification treatment, but cardiac arrest unresponsive to resuscitation maneuvers occurred. Case description and literature review demonstrated that, also in patients without specific risk factors, signs of infection after dog bite should be never underestimated and should be treated with a prompt antibiotic therapy initiation even before occurrence of organ dysfunction.

## 1. Introduction

Dog bites remain a significant cause of morbidity worldwide. Although the exact incidence is uncertain because many victims usually do not refer to emergency department or general practitioner, it is estimated that in the United States approximately 4,5 million people are bitten every year [1]. Lesions caused by bites are mainly lacer wounds requiring reconstructive procedures and often complicated by primary or secondary infections with an incidence ranging from 5 to 18% [2]. Despite being extremely rare, primary infection by* C. canimorsus* after dog bite may be challenging to identify and manage and is rapidly lethal.

## 2. Case Report

**Figure 1:**
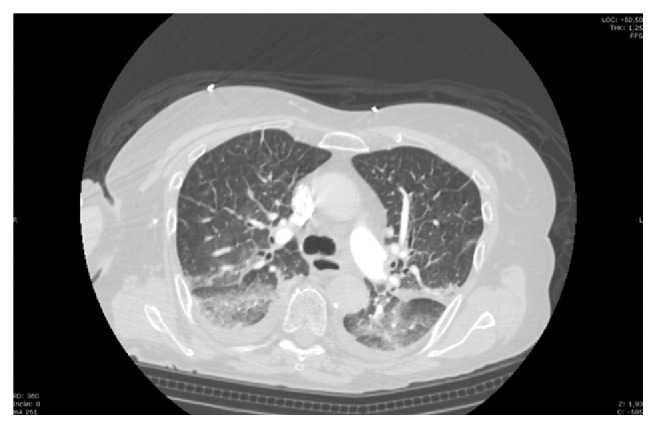
Lung CT scan of the lung.

An 80-year-old woman was accompanied by her son at the emergency department (ED) of our hospital because of progressive altered mental status and persistent high fever in the previous 48 hours. The patient had medical history of depression in treatment with bromazepam and olanzapine. At the ED evaluation, patient was conscious and alert with stable respiratory and hemodynamic conditions and fever (39°C) and mild abdominal pain without defensive reaction. Laboratory tests showed only an increased C-reactive protein (6,1 mg/dl). Abdomen ultrasound showed signs of previous cholecystectomy and a slight dilatation of biliary tree. Two hours later, the patient became progressively drowsy, cyanotic, and mottled on chest and lower extremities despite stable hemodynamic parameters. Arterial blood gas analysis (ABG) revealed mild hypoxia and hypocapnia. Blood and urine samples were collected for microbiological cultures before starting empiric therapy with piperacillin-tazobactam (loading doses 4, 5 g, and 18 g/day continuous infusion). Patient underwent chest and abdomen computed tomography (CT) that showed extended ground-glass area in basal lobes bilaterally (Figure 1) and hypoperfusion in liver, spleen, and kidneys and dilatation of intra- and extrahepatic biliary tree.

**Figure 2:**
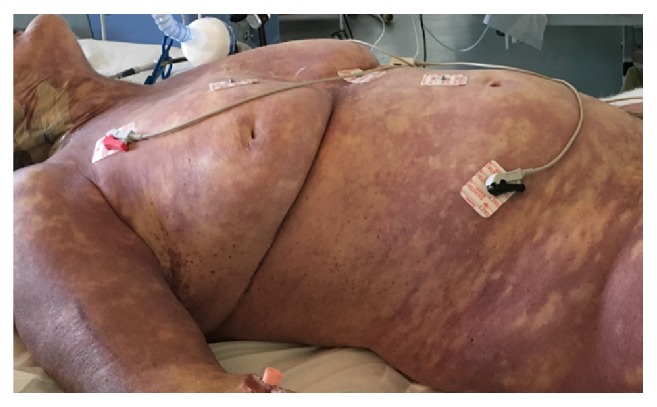
Purpura of trunk and skin.

At the end of CT scan, the patient was transferred to the Intensive Care Unit (ICU) because of severe respiratory failure. At ICU, admission patient was unconscious (Glasgow Coma Scale (GCS) 3/15), hypoxic (SpO2 88% with FiO2 60%), and hypotensive (100/50 mmHg). At physical examination, we observed petechiae and purpura on her trunk and her skin was grayish and mottled (Figure 2). ABG analysis showed a severe metabolic acidosis with lactate 16 mM. The patient became extremely hypotensive and after aggressive fluid resuscitation (40 ml/kg crystalloides) we started norepinephrine (up to 0,2 mcg/Kg/min). Hemodynamic measurements by pulmonary artery catheter revealed a low cardiac Index (CI) (1,3 L/min/m^2^). Echocardiography showed a severe depression of left ventricle ejection fraction without segmental akinesia and, thus, we started dobutamine infusion that after 2 hours was switched to levosimendan because of severe tachycardia without CI improving. At ICU admission, the patient showed severe leukopenia (610/mmc), thrombocytopenia (8000/mmc), and low levels of immunoglobulins (IgG 392 mg/dl, IgA 111 mg/dl and IgM 16 mg/dl); aPTT was undeterminable and INR was prolonged (2,79). Renal and hepatic functional indexes worsened compared to ED first evaluation with a total SOFA score of 22; troponin was high (847 ng/l) as well as procalcitonin (115 ng/ml). The severe hypotension and hyperlactatemia (17 mM) refractory to vasopressors, steroids, and inotropes led us to initiate blood purification (Cytosorb, CytoSorbents Europe GmbH, Germany) as rescue therapy 8 hours after admission. Despite aggressive therapies, cardiac arrest unresponsive to resuscitation maneuvers occurred 4 hours later.

During an extended interview with patient's family members some hours after ICU admission, an important anamnestic element was revealed: 3 days before hospital admission the patient was bitten by a dog, getting a small lesion on the fourth finger of the right hand. Two days after patient's death, the microbiological laboratory reported a blood culture positive for* C. canimorsus* sensitive to any tested antibiotic class. No other microorganisms were identified in the samples evaluated (rectal swab, skin lesions swab, bronchial secretion, urine,* Legionella* and* Streptococcus pneumoniae* antigens, CMV-DNA, and B-D-glucan).

## 3. Discussion

A recent literature review [3] reported 484 cases of infection by* C. canimorsus*, 192 before 1989 and 292 from 1989 to 2014, with a mortality rate of about 25%. Estimated incidence of* C. canimorsus* infection is 0,5-0,7 cases per million people every year. A Dutch study analyzed patients admitted to hospital between 2005 and 2014 with positive blood culture for* C. canimorsus* (65 patients): 24% (16 patients) were treated in ICU because of the presence of severe infections [4].* C. canimorsus* associated mortality depends on the severity of infection with low mortality in meningitis (5%) that increase up to 26-36% in case of bacteremia and sepsis [3, 5, 6] and 60% in patients with septic shock [7], while fulminant purpura has rarely been described [8, 9].

*C. canimorsus* affects humans mainly by dog bites but there are other ways of infection as scratches or close contact with dogs or cats [7, 10]. Despite possible minimal signs at the wound, the bacterium has the property to gain quickly access to blood causing bacteremia [11]. Interaction between Capnocytophaga and immune system is not completely elucidated. It seems that complement system is fundamental in the killing processes of* C. canimorsus* and human sera treated for complement inactivation are not capable of microorganism killing [12]. Incubation period of the pathogen may last from 1 to 7 days. In people with liver disease, immunosuppression, chronic obstructive pulmonary disease, or advance age, the bacterium has a strong pathogenicity and patients may develop severe symptomatic infections such as sepsis, meningitis, septic arthritis, cholecystitis, endocarditis, osteomyelitis, peritonitis, cellulitis, or pneumonia [1, 3, 13–15]. Our patient did not show clinical history at risk for invasive infection, but she was an elderly woman. The progressive impairment of immune system with aging, namely, immune-senescence, is considered one of the main reasons for high infection risk and sepsis complications in elderly population. Clinical studies over the past decade demonstrate unequivocally that sepsis not only causes hyperinflammation but also leads to simultaneous adaptative immune system dysfunction and impaired antimicrobial immunity. Evidence for immunosuppression includes immune cell depletion as in our case report. Nevertheless, it is important to consider that, due to underestimation of the risks related to dog bite, patient came at the hospital and received antibiotics 3 days after the bite and at least 2 days after signs of bacteremia. This delay and the virulence of* C. Canimorsus* may justify the multiple organ dysfunction and septic shock observed in our patient.

Diagnosis of* C. canimorsus* is often difficult because of its slow growth on microbiological media; Gram-stain exam shows, under the microscope, multiple Gram-negative, extracellular, fusiform rods and several intracellular copies of the pathogen in neutrophils. Because of the difficulty for diagnosis, gold standards in detecting the bacterium are molecular tests based on polymerase chain reaction (PCR) amplification and 16S rRNA gene sequencing [5, 7].

The 2014 guidelines for diagnosis and management of skin and soft tissue infections recommend an antibiotic prophylaxis for infections due to dog or cat bites for patients who are immunocompromised, asplenic, or with advanced liver disease; an antimicrobial therapy is also recommended when there is an important local reaction in the site of bite or when the injuries are moderate to severe. If antibiotic is necessary, it should cover aerobic and anaerobic bacteria because of the mixed flora characterizing animal bites.

## 4. Conclusion

In conclusion, the case description and the literature review allowed us to focus on some important aspects. First, dog bite should be never underestimated because severe complications occur even though the patient does not have cellulitis or suppuration on wounds. Second,* C. Canimorsus* may cause invasive infections also in aged patients without specific risk factors due to progressive immune-dysfunction. Last, as for other fulminant infections, the delay between signs of infection and antibiotics should be minimized after dog bite even before the occurrence of organ dysfunction.

## References

[1] Stevens D. L., Bisno A. L., Chambers H. F. (2014). Practice guidelines for the diagnosis and management of skin and soft tissue infections: 2014 update by the infectious diseases society of America. *Clinical infectious diseases: an official publication of the Infectious Diseases Society of America*.

[2] Pfortmueller C. A., Efeoglou A., Furrer H., Exadaktylos A. K. (2013). Dog bite injuries: Primary and secondary emergency department presentations - A retrospective cohort study. *The Scientific World Journal*.

[3] Butler T. (2015). Capnocytophaga canimorsus: an emerging cause of sepsis, meningitis, and post-splenectomy infection after dog bites. *European journal of clinical microbiology & infectious diseases: official publication of the European Society of Clinical Microbiology*.

[4] Hästbacka J., Hynninen M., Kolho E. (2016). Capnocytophaga canimorsus bacteremia: clinical features and outcomes from a Helsinki ICU cohort. *Acta Anaesthesiologica Scandinavica*.

[5] Janda J. M., Graves M. H., Lindquist D., Probert W. S. (2006). Diagnosing Capnocytophaga canimorsus infections. *Emerging Infectious Diseases*.

[6] Le Moal G., Landron C., Grollier G., Robert R., Burucoa C. (2003). Meningitis due to Capnocytophaga canimorsus after receipt of a dog bite: case report and review of the literature. *Clinical infectious diseases: an official publication of the Infectious Diseases Society of America*.

[7] Zajkowska J., Król M., Falkowski D., Syed N., Kamieńska A. (2016). Capnocytophaga canimorsus – an underestimated danger after dog or cat bite – review of literature. *Przegl Epidemiol*.

[8] Cooper J. D., Dorion R. P., Smith J. L. (2015). A rare case of Waterhouse-Friderichsen syndrome caused by Capnocytophaga canimorsus in an immunocompetent patient. *Infection*.

[9] Christiansen C. B., Berg R. M. G., Plovsing R. R., Moller K. (2012). Two cases of infectious purpura fulminans and septic shock caused by Capnocytophaga canimorsus transmitted from dogs. *Scandinavian Journal of Infectious Diseases*.

[10] Valtonen M., Lauhio A., Carlson P. (1995). Capnocytophaga canimorsus septicemia: Fifth report of a cat-associated infection and five other cases. *European Journal of Clinical Microbiology & Infectious Diseases*.

[11] Dedy N. J., Coghill S., Chandrashekar N. K. S., Bindra R. R. (2016). Capnocytophaga canimorsus sepsis following a minor dog bite to the finger: Case report. *Journal of Hand Surgery*.

[12] Zangenah S., Bergman P. (2015). Rapid killing of Capnocytophaga canimorsus and Capnocytophaga cynodegmi by human whole blood and serum is mediated via the complement system. *SpringerPlus*.

[13] Nishioka H., Kozuki T., Kamei H. (2015). Capnocytophaga canimorsus bacteremia presenting with acute cholecystitis after a dog bite. *Journal of Infection and Chemotherapy*.

[14] Oehler R. L., Velez A. P., Mizrachi M., Lamarche J., Gompf S. (2009). Bite-related and septic syndromes caused by cats and dogs. *The Lancet Infectious Diseases*.

[15] Pers C., Gahrn-Hansen B., Frederiksen W. (1996). Capnocytophaga canimorsus septicemia in Denmark, 1982-1995: Review of 39 cases. *Clinical Infectious Diseases*.

